# Neonatal Growth, Nutrition, and Neurodevelopment: A Complex Relationship

**DOI:** 10.3390/nu15214634

**Published:** 2023-10-31

**Authors:** Julián Rodríguez-Suárez, Gonzalo Solís-Sánchez, Isolina Riaño-Galán

**Affiliations:** AGC Pediatría, Hospital Universitario Central de Asturias, Instituto Investigación Sanitaria Principado de Asturias (ISPA), Universidad de Oviedo, 33011 Oviedo, Spain; rodriguez.julian@uniovi.es (J.R.-S.); rianoisolina@uniovi.es (I.R.-G.)

Growth in the neonatal period is critical for the neurodevelopment of the individual, both in low- and middle-income countries. This occurs in healthy neonates and, above all, in the case of premature babies or neonates with pathologies that may interfere with their growth for any reason. An appropriate supply of macro- and micronutrients is essential at the end of the evolution of immature organs awaiting the completion of their development after birth and the brain is, without a doubt, the most important of them all [1,2,3,4,5].

In this Special Issue, we want to review the key aspects, tools, and considerations to evaluate the relationships between neurodevelopment, nutrition, and growth in the first phase of life. Our goal is to present the evidence and identify gaps in the influence of different factors on the growth and neurodevelopment of the child, with special emphasis on both maternal and neonatal nutritional aspects.

The brain needs optimal nutrition to achieve its goals as it is a metabolically very active organ that consumes a significant proportion of nutrient and energy intake [6]. Therefore, nutrition in the last phase of fetal development and the neonatal age can critically impact the cognitive abilities and mental development of the future adult. An association has been demonstrated between nutrition, weight gain, and brain volume with white matter maturation and neurodevelopment in different populations [2,7]. Currently, efforts are being made to optimize this nutrition, focusing on growth factors and micro- and macro-nutrients, which are critical for development at this stage of life [8].

Appropriate maternal nutrition during pregnancy facilitates, although it does not ensure, birth with the adequate growth and development of the newborn. A significant association has been found between the nutritional status and health of mother and child and maternal educational attainment through assessing the development at 15 months of Tanzanian children using Bayley Scales of Infant and Toddler Development [9]. On the contrary, mothers’ malnutrition, regardless of the cause, causes the child’s future to be compromised. Malnourished mothers due to a lack of nutrition or due to their pathologies (eating behavior disorders; stress; chronic, infectious, inflammatory, or oncological processes; inadequate disease treatment) can cause the suboptimal growth and development of the fetus and, consequently, the future child’s neurodevelopment [10,11,12,13]. There is no doubt that, in countries or environments with low resources, early interventions for pregnant mothers are essential to improve the long-term health of their children [14]. This aspect is clear in the article by Thair et al. on the effect of micronutrient supplementation on pre-pregnant mothers and the effects on their children at 6 months [15].

Along with the old global nutrition problems in countries with low socio-economic levels, there are also new maternal pathological situations in more developed countries with higher incomes. Eating behavior problems or exclusion diets, so common in the so-called developed world, are still serious causes of malnutrition that can endanger the neurodevelopment of future children [16].

It is well known that, after birth, the rapid brain development in the first two years makes this stage of life one of the most sensitive and vulnerable to nutrition problems due to its high requirements. Thus, if the baby and its mother do not present any pathology, nutrition with exclusive breastfeeding will be sufficient for adequate growth and neurodevelopment during the first six months of life [17,18]. However, any deviation from this idyllic situation may affect the newborn’s growth in different ways, by excess or by defect.

This is the case of babies born prematurely; the birth of a very premature child is a major problem in terms of nutritional deviation. The arrival of a neonate without completing the third trimester of gestation makes the risk of malnutrition, growth impairment, and the alteration of subsequent neurodevelopment common, despite the improvements in nutrition techniques in recent years. The concern regarding the optimal integral development and growth in these very premature babies is increasing, and efforts are being made to achieve high-quality parental feedings or to stimulate breastfeeding in premature babies, either with their own mother’s milk or donated fortified milk from a bank, according to their basic needs [4,19,20]. However, in addition to nutrition, it is essential to address other factors, both neonatal and post-neonatal, and promote understanding of the stimulation and prevention of disability.

We are not only talking about malnutrition by default but also by excess. The difficulties also lie in knowing what the optimum growth is for these children (there are different definitions for talking about adequate extrauterine growth), what the goal is, and how to achieve this optimal growth [21,22]. The most important thing is to know what contributions of macro- and micro-nutrients, in terms of both quality and quantity, are adequate to reach these without putting the children at subsequent metabolic risk. In extreme preterms, there currently seems to be a line of knowledge that maintains an influence between a greater protein intake and the beneficial effects on growth and neurodevelopment, but it is also advisable to focus on the balance of contributions given the existing risk of refeeding syndrome [23,24].

The importance of nutrition, growth, and neurodevelopment goes far beyond maternal malnutrition and very premature babies. There are many other causes of possible malnutrition and poor growth and neurodevelopment in neonates [25]. There is a complex relationship between nutrition, environmental factors (social, physical, etc.), inflammation, genetic heterogeneity, and neurodevelopment. Epigenetic factors may play a prominent role, producing an imprint of the genome in the early stages of life regarding later susceptibility to developmental alterations. Also, environmental toxins, such as endocrine disruptors, play an important role in this field [26]. We can say that children with an adequate nutritional status will probably reach their full development if we take care of their nutrition, as well as their physical and mental environment [27].

On the other hand, gestation and the first years of life represent a dynamic, plastic period for intestinal colonization regarding the microbiota and for brain development. The type of nutrition is essential in this colonization and the subsequent health of the children, including their growth and neurodevelopment. The existence of a complex “dialogue” between the intestinal microbiota and the nervous system, and the brain in particular, has been suggested [28,29,30,31,32].

We conclude by stating that the nutritional status of the pregnant mother, her diet, including macro- and micro-nutrients, and nutrition in the early neonatal period and the first two years of life, have an essential impact on growth and neurodevelopment. Although the intrinsic mechanisms are not yet very clear, and although other genetic, epigenetic, and environmental factors play an important role in this nutrition–neurodevelopment interrelation, taking care of nutrition is essential. In the future, personalized nutrition, through new data sciences, will possibly play a key role in improving these problems [2].

## References

[1] Cusick S.E., Georgieff M.K. (2016). The role of nutrition in brain development: The golden opportunity of the “First 1000 Days.”. J. Pediatr..

[2] Morton S.U., Leyshon B.J., Tamilia E., Vyas R., Sisitsky M., Ladha I., Lasekan J.B., Kuchan M.J., Grant P.E., Ou Y. (2022). A Role for Data Science in Precision Nutrition and Early Brain Development. Front. Psychiatry.

[3] Babikako H.M., Bourdon C., Mbale E., Aber P., Birabwa A., Chimoyo J., Voskuijl W., Kazi Z., Massara P., Mukisa J. (2022). Neurodevelopment and Recovery From Wasting. Pediatrics.

[4] Lockyer F., McCann S., Moore S.E. (2021). Breast Milk Micronutrients and Infant Neurodevelopmental Outcomes: A Systematic Review. Nutrients.

[5] Ottolini K.M., Andescavage N., Keller S., Limperopoulos C. (2020). Nutrition and the developing brain: The road to optimizing early neurodevelopment: A systematic review. Pediatr. Res..

[6] Goyal M.S., Iannotti L.L., Raichle M.E. (2018). Brain nutrition: A life span approach. Annu. Rev. Nutr..

[7] Coviello C., Keunen K., Kersbergen K.J., Groenendaal F., Leemans A., Peels B., Isgum I., Viergever M.A., de Vries L.S., Buonocore G. (2018). Effects of early nutrition and growth on brain volumes, white matter microstructure, and neurodevelopmental outcome in preterm newborns. Pediatr. Res..

[8] Chan A.P., Rostas S., Rogers S., Martin C.R., Calkins K.L. (2023). Parenteral Nutrition in the Neonatal Intensive Care Unit: Intravenous Lipid Emulsions. Clin. Perinatol..

[9] Blakstad M.M., Smith E.R., Etheredge A., Locks L.M., McDonald C.M., Kupka R., Kisenge R., Aboud S., Bellinger D., Sudfeld C.R. (2019). Nutritional, Socioeconomic, and Delivery Characteristics Are Associated with Neurodevelopment in Tanzanian Children. J. Pediatr..

[10] Suchdev P.S., Boivin M.J., Forsyth B.W., Georgieff M.K., Guerrant R.L., Nelson C.A. (2017). Assessment of Neurodevelopment, Nutrition, and Inflammation From Fetal Life to Adolescence in Low-Resource Settings. Pediatrics.

[11] Bhutta Z.A., Guerrant R.L., Nelson C.A. (2017). Neurodevelopment, Nutrition, and Inflammation: The Evolving Global Child Health Landscape. Pediatrics.

[12] Vakil P., Henry A., Craig M.E., Gow M.L. (2022). A review of infant growth and psychomotor developmental outcomes after intrauterine exposure to preeclampsia. BMC Pediatr..

[13] Vohr B.R., Poggi Davis E., Wanke C.A., Krebs N.F. (2017). Neurodevelopment: The Impact of Nutrition and Inflammation During Preconception and Pregnancy in Low-Resource Settings. Pediatrics.

[14] Crawford M.A., Wang Y., Marsh D.E., Johnson M.R., Ogundipe E., Ibrahim A., Rajkumar H., Kowsalya S., Kothapalli K.S.D., Brenna J.T. (2022). Neurodevelopment, nutrition, and genetics. A contemporary retrospective on neurocognitive health on the occasion of the 100th anniversary of the National Institute of Nutrition, Hyderabad, India. Prostaglandins Leukot Essent. Fatty Acids.

[15] Thahir A.I.A., Li M., Holmes A., Gordon A. (2023). Exploring Factors Associated with Stunting in 6-Month-Old Children: A Population-Based Cohort Study in Sulawesi, Indonesia. Nutrients.

[16] Marshall N.E., Abrams B., Barbour L.A., Catalano P., Christian P., Friedman J.E., Hay W.W., Hernandez T.L., Krebs N.F., Oken E. (2022). The importance of nutrition in pregnancy and lactation: Lifelong consequences. Am. J. Obstet. Gynecol..

[17] Deoni S., Dean D., Joelson S., O’Regan J., Schneider N. (2018). Early nutrition influences developmental myelination and cognition in infants and young children. Neuroimage.

[18] Gilbreath D., Hagood D., Alatorre-Cruz G.C., Andres A., Downs H., Larson-Prior L.J. (2023). Effects of Early Nutrition Factors on Baseline Neurodevelopment during the First 6 Months of Life: An EEG Study. Nutrients.

[19] Hair A.B., Scottoline B., Good M. (2023). Dilemmas in human milk fortification. J. Perinatol..

[20] Elliott M.J., Golombek S.G. (2022). Evolution of Preterm Infant Nutrition from Breastfeeding to an Exclusive Human Milk Diet: A Review. Neoreviews.

[21] González-García L., Mantecón-Fernández L., Suárez-Rodríguez M., Arias-Llorente R., Lareu-Vidal S., Ibáñez-Fernández A., Caunedo-Jiménez M., González-López C., Fernández-Morán E., Fernández-Colomer B. (2022). Postnatal Growth Faltering: Growth and Height Improvement at Two Years in Children with Very Low Birth Weight between 2002-2017. Children.

[22] González García L., García López E., Fernández Colomer B., Mantecón Fernández L., Lareu Vidal S., Suárez Rodríguez M., Arias Llorente R., Solís Sánchez G. (2022). Growth outcome at 2 years using Fenton and Intergrowth-21st charts in infants less than 1500 g. An. Pediatr..

[23] Cormack B.E., Harding J.E., Miller S.P., Bloomfield F.H. (2019). The Influence of Early Nutrition on Brain Growth and Neurodevelopment in Extremely Preterm Babies: A Narrative Review. Nutrients.

[24] Bloomfield F.H., Cormack B.E. (2021). Strategies in Neonatal Care to Promote Growth and Neurodevelopment of the Preterm Infant. Nestle Nutr. Inst. Workshop Ser..

[25] Trivedi A., Browning Carmo K., Jatana V., James-Nunez K., Gordon A. (2023). Growth and risk of adverse neuro-developmental outcome in infants with congenital heart disease: A systematic review. Acta Paediatr..

[26] Schug T.T., Blawas A.M., Gray K., Heindel J.J., Lawler C.P. (2015). Elucidating the links between endocrine disruptors and neurodevelopment. Endocrinology.

[27] Mattei D., Pietrobelli A. (2019). Micronutrients, and Brain Development. Curr. Nutr. Rep..

[28] Codagnone M.G., Stanton C., O’Mahony S.M., Dinan T.G., Cryan J.F. (2019). Microbiota and Neurodevelopmental Trajectories: Role of Maternal and Early-Life Nutrition. Ann. Nutr. Metab..

[29] Lima-Ojeda J.M., Rupprecht R., Baghai T.C. (2017). «I Am I and My Bacterial Circumstances»: Linking Gut Microbiome, Neurodevelopment, and Depression. Front. Psychiatry.

[30] Carlson A.L., Xia K., Azcarate-Peril M.A., Goldman B.D., Ahn M., Styner M.A., Thompson A.L., Geng X., Gilmore J.H., Knickmeyer R.C. (2018). Infant Gut Microbiome Associated With Cognitive Development. Biol. Psychiatry.

[31] Panchal H., Athalye-Jape G., Rao S., Patole S. (2023). Growth and neuro-developmental outcomes of probiotic supplemented preterm infants. A systematic review and meta-analysis. Eur. J. Clin. Nutr..

[32] Mathews T., Hayer S.S., Dinkel D., Hanish A., Poppert Cordts K.M., Rasmussen H., Moore T. (2023). Maternal-Child Microbiome and Impact on Growth and Neurodevelopment in Infants and Children: A Scoping Review. Biol. Res. Nurs..

